# NAD^+^ Metabolism Reprogramming Drives SIRT1‐Dependent Deacetylation Inducing PD‐L1 Nuclear Localization in Cervical Cancer

**DOI:** 10.1002/advs.202412109

**Published:** 2025-02-23

**Authors:** Xinyi Lu, Pingping Jin, Qianyun Tang, Min Zhou, Hanjie Xu, Chen Su, Lei Wang, Feng Xu, Min Zhao, Yongxiang Yin, Jinqiu Zhang, Zhen Jia, Xinrui Peng, Jie Zhou, Lu Wang, Yan Chen, Min Wang, Min Yang, Daozhen Chen, Yu Chen

**Affiliations:** ^1^ Affiliated Women's Hospital of Jiangnan University Jiangnan University Jiangsu 214002 China; ^2^ Wuxi Medical Center Nanjing Medical University Jiangsu 214023 China; ^3^ Department of Hepatopancreatobiliary Surgery Jiangnan University Medical Center Jiangsu 214002 China; ^4^ Department of Laboratory Haidong Second People's Hospital Haidong 810699 China; ^5^ Molecular Imaging Centre Jiangsu Institute of Nuclear Medicine Jiangsu 214063 China

**Keywords:** acetyl‐proteomics, cervical cancer, immunotherapy resistance, NAD^+^ metabolism, PD‐L1, SIRT1

## Abstract

Cervical cancer (CC) is a major health threat to women, with immunotherapies targeting the programmed death receptor 1/programmed death ligand 1(PD‐1/PD‐L1) axis showing promise but encountering resistance in a significant patient population. This resistance has driven a critical quest to uncover the underlying mechanisms. This study uncovers a novel metabolic axis involving the nicotinamide adenine dinucleotide (NAD^+^) salvage pathway enzyme nicotinamide phosphoribosyltransferase (NAMPT) and the deacetylase Sirtuin 1 (SIRT1), which regulates PD‐L1 expression and nuclear localization in CC. This axis may be a key factor contributing to the resistance observed in immunotherapy. This study reveals that PD‐L1 overexpression in cancers is regulated by both transcriptional and post‐transcriptional processes. Acetyl‐proteomic analysis pinpoints SIRT1 as a central regulator in the deacetylation of histone H3 at lysines 27, which may influence PD‐L1 subcellular distribution. This finding reveals the epigenetic control of immune checkpoint proteins by metabolic pathways, offering a new perspective on the regulation of PD‐L1. The identification of the NAMPT/SIRT1 metabolic axis as a critical factor suggests that targeting this axis may enhance therapeutic responses.

## Graphical abstract

The identification of PD‐L1 localization may be a key factor in the resistance to immunotherapy observed in patients. The discovery that PD‐L1 over expression is regulated by both transcriptional and post‐transcriptional processes. The acetyl‐proteomic analysis that identified SIRT1 as a key player in the deacetylation of histone H3 at lysines 27 and 36, is a critical step for PD‐L1 nuclear repositioning.

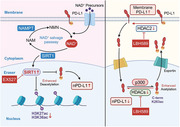

## 1 Introduction

Cervical cancer (CC), a leading cause of cancer‐related deaths among women,^[^ ^1^, ^2^ ^]^ presents a challenge to immunotherapy because of the sophisticated mechanisms of immune evasion of the tumors.^[^ ^3^, ^4^, ^5^ ^]^ Despite the transformative impact of immune checkpoint inhibitors targeting the PD‐1/PD‐L1 axis in cancer treatment, their efficacy in CC is less than excellent.^[^ ^6^, ^7^ ^]^ A key player in immune evasion is the overexpression of PD‐L1, which interacts with the PD‐1 receptor on T cells, thereby inhibiting their activation and allowing tumor cells to sidestep immune destruction.^[^ ^8^, ^9^ ^]^ Emerging evidence suggests that the role of PD‐L1 extends beyond the cell surface, with potential nuclear localization, which adds a layer of complexity to its function and raises critical questions regarding its contribution to tumor progression and resistance to immunotherapy.^[^ ^10^, ^11^, ^12^ ^]^ Consequently, unraveling the regulation of PD‐L1 expression and its localization is essential for developing potent immunotherapeutic strategies against CC.

Aberrant NAD^+^ metabolism has been increasingly recognized as a pivotal factor in cancer progression and metastasis.^[^ ^13^, ^14^ ^]^ We have previously reported aberrant NAD^+^ in the tumor microenvironment (TME), suggesting a link between metabolic reprogramming and CC.^[^ ^15^ ^]^ NAD^+^ is crucial for cellular processes, such as energy production, DNA repair, and cellular signaling, and is often dysregulated in cancer cells.^[^ ^16^, ^17^, ^18^ ^]^ Enzymes like NAMPT, involved in NAD^+^ biosynthesis, are frequently upregulated to satisfy the intense metabolic demands of rapidly dividing cells.^[^ ^13^, ^19^ ^]^ This metabolic reprogramming not only sustains cancer cell survival but also fosters metastasis by modulating pathways that promote invasion and immune evasion.^[^ ^20^ ^]^ In CC, dysregulated NAD^+^ metabolism is linked to the facilitation of immune escape mechanisms that impede effective immunotherapy.

Posttranslational modifications (PTMs) of PD‐L1 modulate its stability and function. Besides, nuclear PD‐L1 (nPD‐L1) has been correlated with the regulation of gene expression in relation to immune suppression, adding a layer of complexity to its role in tumor immunity.^[^ ^20^ ^]^ PTMs, including phosphorylation, glycosylation, ubiquitination, and acetylation, can modulate PD‐L1's stability, localization, and interactions with other molecules.^[^ ^21^, ^22^, ^23^ ^]^ The presence of nPD‐L1 has been associated with the regulation of gene expression linked to immune suppression, introducing an additional layer of complexity to its role in tumor immunity.^[^ ^24^ ^]^ These modifications can significantly affect PD‐L1's stability, localization, and interactions with other molecules. For instance, Casitas B‐lineage lymphoma family proteins have been shown to ubiquitinate PD‐L1, influencing its degradation and surface expression.^[^ ^25^ ^]^ Additionally, the role of glycosylation in PD‐L1 stability has been elucidated, with enzymes such as STT3 and B3GNT3 contributing to PD‐L1 glycosylation and protection from degradation.^[^ ^26^ ^]^ Determining the posttranslational regulation of PD‐L1 is vital for identifying novel therapeutic targets that can enhance the efficacy of immune checkpoint blockade in CC.

Our study builds upon this understanding by investigating how NAD^+^ metabolism influences PD‐L1 posttranslational acetylation and subsequent nuclear localization, which may contribute to immune evasion and therapy resistance in CC. Deacetylation, a key PTM influenced by NAD^+^ metabolism, is mediated by SIRT1, which is known to remove acetyl groups from both histones and non‐histone proteins, including PD‐L1.^[^ ^27^ ^]^ SIRT1‐mediated deacetylation can reshape PD‐L1 cellular localization, promoting its nuclear translocation and contributing to immune evasion. Histone deacetylation by SIRT1 has been implicated in the repression of immune‐related genes,^[^ ^28^ ^]^ suggesting a potential link between NAD^+^ metabolism, histone modification, and PD‐L1‐mediated immune escape. However, the precise molecular mechanisms linking these processes are not well defined.

In this study, we explored the role of NAD^+^ metabolism in the regulation of PD‐L1 expression and nuclear localization in cervical cancer, with a spotlight on the NAMPT/SIRT1 axis. We demonstrated that NAMPT‐mediated NAD^+^ biosynthesis induces deacetylation of histone H3 at Lys27 (H3K27), which may be associated with PD‐L1 and bolsters tumor immune evasion. Our findings revealed a novel metabolic‐epigenetic pathway that governs PD‐L1 regulation, which may be used in the development of potential therapeutic interventions to enhance the efficacy of immunotherapy in CC.

## 2 Results

### 2.1 PD‐L1 is Upregulated in Patients with CC and Shows Nuclear Localization

**Figure 1:**
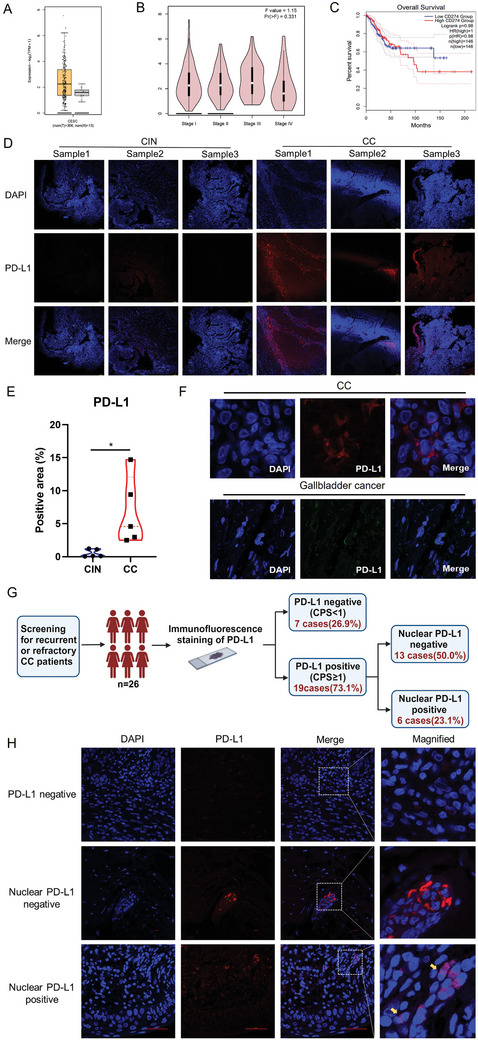
PD‐L1 is upregulated in cervical cancer patients and shows nuclear localization. A) PD‐L1 expression in CC patients (n = 306) and healthy people (n = 13) from the CESC dataset. B) The violin plot of the correlation of PD‐L1 expression and tumor stage in human CC tissues from the TCGA dataset. C) Kaplan–Meier analysis of overall survival (OS) in CC patients (n = 146 pairs) according to the expression of PD‐L1. D) Representative images of IF staining of PD‐L1 in CIN and CC samples (n = 3). Scale bars, 100 µm. E) Statistical results of the PD‐L1 positive area in CIN and CC samples (n = 5). Data are presented as means ± SD. Two‐tailed unpaired Student's t‐tests. ^*^ *p* < 0.05. F) Representative images of IF staining of nuclear PD‐L1 in CC and Gallbladder cancer samples. Scale bars, 10 µm. G) Flow diagram for the identification of PD‐L1 expression and subcellular localization in CC patients (n = 26). H) Representative images of IF staining of PD‐L1 expression in CC patients. Scale bars, 10 µm.

To investigate the role of PD‐L1 in CC progression, we first analyzed its expression in the TCGA dataset. PD‐L1 expression was significantly upregulated in patients with CC (n = 306) compared to that in normal and GTEx tissues (n = 13) (**Figure** **1**A). These findings are consistent with those of previous reports showing PD‐L1 overexpression in various malignancies, including lung^[^ ^29^, ^30^, ^31^ ^]^ and breast cancers,^[^ ^32^, ^33^, ^34^ ^]^ where it was correlated with immune evasion and poor prognosis. Furthermore, we observed a positive correlation between PD‐L1 expression and tumor stage (Figure 1B), with patients exhibiting higher PD‐L1 levels tending to have a worse prognosis (Figure 1C). These observations align with prior studies demonstrating that advanced‐stage cancers are often associated with elevated PD‐L1 levels.^[^ ^35^, ^36^ ^]^

Next, we performed immunofluorescent (IF) staining of cervical intraepithelial neoplasia (CIN) and CC tissues. PD‐L1 expression was markedly higher in CC tissues than in CIN tissues (Figure 1D). Quantitative analysis revealed that the PD‐L1‐positive area in CC tissues was ≈6.8%, whereas that in CIN tissues was only 0.5% (Figure 1E). In a larger cohort of 26 patients with CC, 73.1% (19/26) exhibited PD‐L1 positivity (CPS ≥ 1) (Figure 1G).

Subcellular localization of PD‐L1 has been increasingly recognized as a key factor affecting the response to PD‐1/PD‐L1 blockade therapy.^[^ ^11^, ^37^, ^38^ ^]^ In this study, we observed that, while the majority of PD‐L1 was localized to the cell membrane in CC tissues, a significant proportion was also present in the nucleus (Figure 1F,H). Nuclear localization of PD‐L1 was observed in 31.6% (6/19) of PD‐L1‐positive CC cases (Figure 1G), a finding that differs from that of gallbladder cancer, where PD‐L1 is predominantly membrane‐bound (Figure 1F). Significantly, our analysis revealed that 66.7% (4/6) of patients exhibiting nPD‐L1 positivity demonstrated resistance to immune therapy. This correlation strongly implies that the nuclear localization of PD‐L1 may be a key factor contributing to ineffectual treatments.^[^ ^10^, ^39^ ^]^ Together, these data suggest that PD‐L1 is not only upregulated in CC but also exhibits nuclear localization, which may contribute to malignant progression and potentially influence immune escape.

### 2.2 NAMPT‐Mediated NAD^+^ Metabolism Drives PD‐L1 Expression in CC Cells

**Figure 2:**
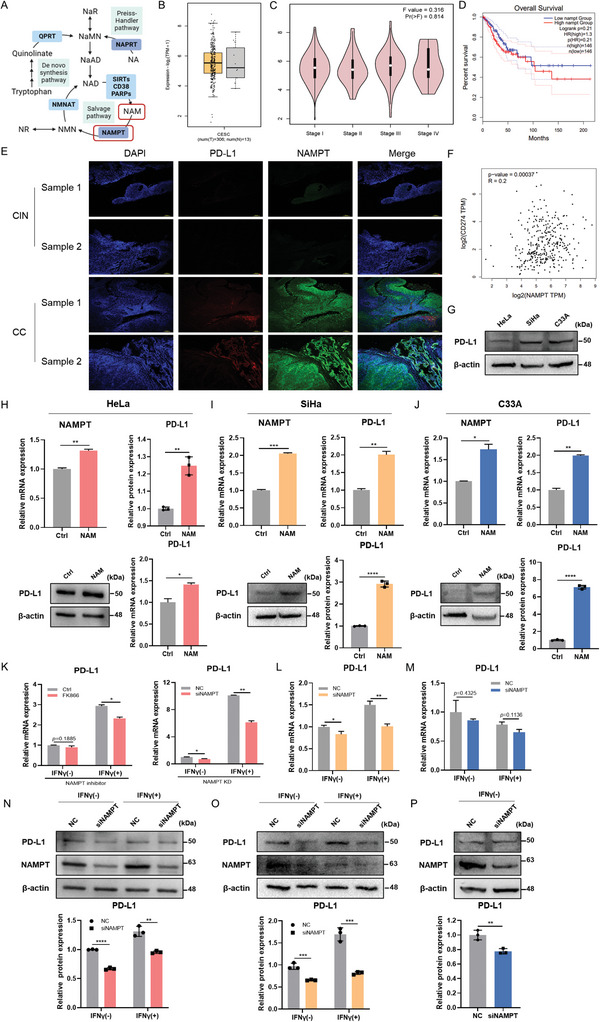
NAMPT‐mediated NAD^+^ metabolism drives PD‐L1 expression in CC cells. A) Schematic diagram of NAD^+^ biosynthesis. B) Nampt expression in CC patients (n = 306) and healthy people (n = 13) from the CESC dataset. C) The violin plot of the correlation of Nampt expression and tumor stage in human CC tissues from the TCGA dataset. D) Kaplan–Meier analysis of OS in CC patients (n = 292) according to the expression of Nampt. E) Representative images of IF staining of PD‐L1 and NAMPT in CIN and CC samples (n = 2). Scale bars, 100 µm. F) Correlation analysis of Nampt and PD‐L1 mRNA expression in human CC tissues. G) The protein levels of PD‐L1 in HeLa, SiHa, and C33A cell lines. H–J) PD‐L1 and Nampt mRNA levels and PD‐L1 protein levels in HeLa (H), SiHa (I), and C33A (J) with NAD^+^ precursor NAM (1mm) treatment. K) PD‐L1 mRNA levels in HeLa with Nampt knockdown (KD), Nampt inhibitor (FK866) (5 µM) pretreatment upon or without IFNγ (10ng mL^−1^) stimulation. L,M) PD‐L1 mRNA levels in SiHa (L) and C33A (MCC) with Nampt KD upon or without IFNγ stimulation. N–P) The protein levels of PD‐L1 and NAMPT in HeLa (N), SiHa (O), and C33A (P) with Nampt KD. Data were represented as mean ± SD. Two‐tailed unpaired Student's t‐tests. ^*^ *p* < 0.05, ^**^ *p *< 0.01, ^***^ *p* < 0.001, ^****^ *p* < 0.0001.

Our previous study demonstrated that NAD^+^ metabolism in the TME is aberrant, suggesting a metabolic basis for CC pathogenesis. NAMPT is the limiting enzyme in the NAD^+^ synthesis pathway and is crucial for maintaining NAD^+^ pool levels (**Figure** **2**A). In addition, by comparing NAMPT and nicotinic acid phosphoribosyltransferase (NAPRT; another enzyme responsible for NAD^+^ synthesis) expression levels, we determined that NAPRT showed no significant association with PD‐L1(Figure S1A–C, Supporting Information). To investigate the role of NAMPT‐mediated NAD^+^ metabolism in PD‐L1 expression in CC, we analyzed the expression of *Nampt* in CC (n = 306) and found the expression is higher than that in normal and adjacent tissues in the TCGA and GTEx datasets (n = 13) (Figure 2B). Similar to that of PD‐L1, NAMPT expression correlated with tumor stage, and high NAMPT levels were associated with poor survival (Figure 2C,D). This parallels previous studies on other cancers, such as colorectal^[^ ^40^, ^41^ ^]^ and breast cancers,^[^ ^42^, ^43^ ^]^ in which NAMPT has been implicated in promoting tumor progression through NAD^+^‐dependent pathways.

To further explore the relationship between NAMPT and PD‐L1, a modest correlation with a significant p‐value between *Nampt* and PD‐L1 mRNA expression (r = 0.2, *p* < 0.001) was confirmed in a cervical squamous cell carcinoma dataset (Figure 2F). Moreover, in vitro experiments supported this association, given that HeLa cells treated with nicotinamide (NAM), a NAD^+^ precursor, showed a notable increase in PD‐L1 expression at both mRNA and protein levels (Figure 2H). Conversely, NAMPT inhibition using small interfering RNA (siRNA) or FK866 (an inhibitor of NAMPT) reduced PD‐L1 expression. Interestingly, the regulation of PD‐L1 by NAMPT was enhanced when cells were stimulated with interferon‐gamma (IFNγ), an established activator of PD‐L1 (Figure 2K,N).

To elucidate the mechanisms underlying CC, we extended our investigation to include SiHa and C33A cell lines and compared the results with those of the HeLa cell line. We found that the expression levels of PD‐L1 in SiHa and C33A cells were higher than in HeLa cells (Figure 2G). However, upon exposure to NAM, both SiHa and C33A cells also exhibited an upregulation of PD‐L1 expression at the transcriptional and protein levels (Figure 2I,J). This response was attenuated by *Nampt* knockdown (KD), suggesting a direct link between NAMPT activity and PD‐L1 expression. Further analysis with IFNγ revealed a cell line‐specific response. SiHa cells mirrored the HeLa cell line's response to IFNγ, whereas C33A cells displayed no significant increase in PD‐L1 expression despite IFNγ stimulation. However, *Nampt* KD consistently reduced PD‐L1 levels in C33A cells (Figure 2L,M,O,P; Figure S1D,E, Supporting Information). Collectively, these observations underscore the pivotal role of *Nampt* in the regulation of PD‐L1 expression in CC cells, potentially offering a novel therapeutic target for modulating PD‐L1 levels and enhancing the efficacy of immunotherapies.

### 2.3 NAD^+^ Metabolism Promotes Nuclear PD‐L1 Localization in CC In Vivo

**Figure 3:**
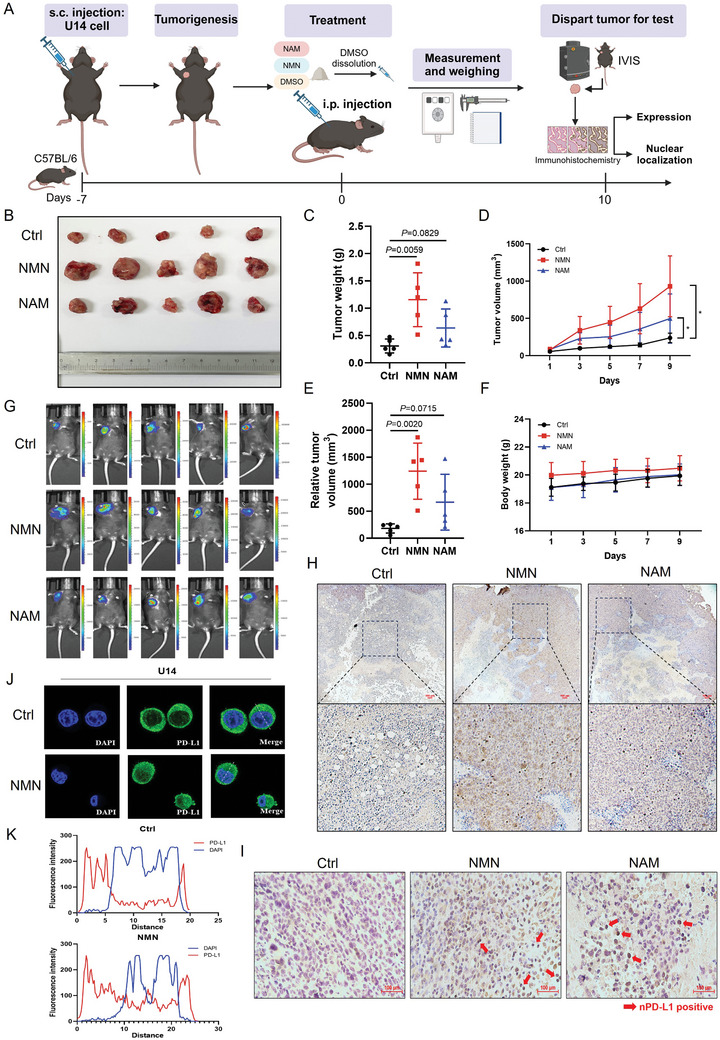
NAD^+^ metabolism promotes nuclear PD‐L1 localization in CC in vivo. A) Scheme representing the experimental procedure. B–F) Tumor weight (C), relative tumor volume (D), tumor growth curves (E) and body weight (F) of C57BL/6 mice injected subcutaneously with U14 cells with NMN or NAM or solvent control (300mg kg^−1^) (n = 5). Data are presented as means ± SD. Two‐tailed unpaired Student's t‐tests. ^*^ *p* < 0.05. G) IVIS images of mice treated with NMN, NAM or solvent at day 10 post‐injection. H) Representative immunohistochemical (IHC) staining of PD‐L1 expression of tumor tissues in mice treated with NMN, NAM, or solvent. Scale bars, 100 µm. I) Representative IHC staining of nuclear PD‐L1 expression of tumor tissues in mice treated with NMN, NAM, or solvent. Scale bars, 100 µm. J,K) Representative images of IF staining of PD‐L1 (J) and its fluorescence intensity (K) of U14 treated with NMN or control (1mm).

To confirm the role of NAD^+^ metabolism in the regulation of PD‐L1 expression in CC in vivo, we constructed a subcutaneous tumor model in C57BL/6 mice using U14‐luc cells (**Figure** **3**A). All mice developed comparable‐sized tumors, showing similar weight changes (Figure 3B,F). Compared to the solvent control group, treatment with either the NAD^+^ precursor nicotinamide mononucleotide (NMN) or NAM significantly promoted tumor growth in mice. There were significant differences in tumor weight and tumor volume between the mice treated with NMN or NAM and the control group (Figure 3C–E). The results were analyzed by measuring bioluminescence using an IVIS imaging system (Figure 3G). Subsequently, to confirm the protein levels of PD‐L1 in the tumor tissues, we performed immunohistochemical (IHC) staining. We determined that PD‐L1 protein levels were markedly elevated in mice treated with NMN or NAM compared with those in the control group (Figure 3H). In addition, the number of nuclear PD‐L1 positive cells significantly increased in tumors of mice treated with NAM or NMN (Figure 3I), which is consistent with our previous experimental results. We further validated the nuclear localization at the cellular level. Compared with the control, the nuclear PD‐L1 fluorescence intensity of U14 cells significantly increased after NMN treatment, consistent with the IHC results (Figure 3J,K). Therefore, our results demonstrated that NAD^+^ metabolism drives PD‐L1‐mediated immune escape in CC.

### 2.4 NAMPT Enhances the Nuclear Localization of PD‐L1 in CC Cells

**Figure 4:**
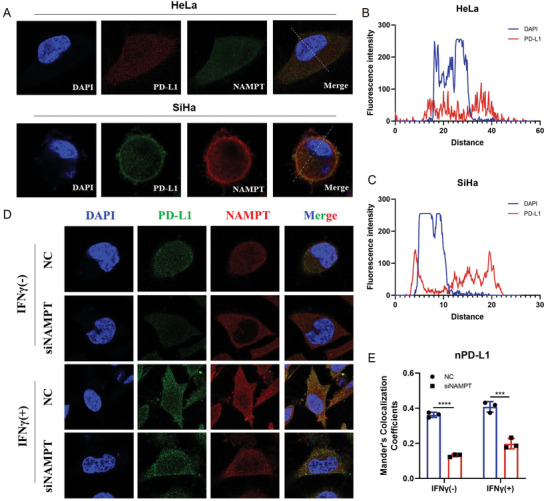
NAMPT enhances the nuclear localization of PD‐L1 in CC cells. A–C) Representative images of IF staining of PD‐L1 and NAMPT in HeLa and SiHa (A). HeLa, scale bars, 50 µm. SiHa, scale bars, 10 µm. Colocalization analysis of PD‐L1 and DAPI of (A) by ImageJ software (B,C). D, E) Representative images of IF staining of NAMPT and PD‐L1 in HeLa with Nampt KD pretreatment upon or without IFNγ stimulation (D). Scale bars, 20µm. Mander's Colocalization Coefficients of PD‐L1 and DAPI (n = 3) (E). Data are presented as means ± SD. Two‐tailed unpaired Student's t‐tests. ^***^ *p* < 0.001, ^****^ *p* < 0.0001.

To investigate the role of NAD^+^ metabolism in regulating PD‐L1 nuclear localization, we performed IF staining for NAMPT and PD‐L1 in HeLa cells. Interestingly, we found PD‐L1 co‐localized with NAMPT. The localization of PD‐L1 in HeLa cells was not limited to the membrane, and a large proportion of PD‐L1 was localized within the nucleus (**Figure** **4**A,B). Furthermore, in SiHa cells, PD‐L1 was mostly localized on the membrane, but was also expressed in the nucleus (Figure 4A,C), which is consistent with the IF results from CC tissues (Figure 1H). Though the protein level of PD‐L1 in HeLa cells was lower than that in SiHa cells (Figure 2G), HeLa cells showed higher expression of nPD‐L1. Notably, nPD‐L1 has been reported to play an oncogenic role independent of immune checkpoint regulation, suggesting the nuclear localization of PD‐L1 in HeLa cells may be related to adverse therapeutic effects.^[^ ^44^, ^45^ ^]^

Subsequently, we investigated the expression of PD‐L1 in Hela cells after Nampt KD. First, we demonstrated that *Nampt* inhibition had no significant effect on proliferating cell nuclear antigen (PCNA) at the protein level (Figure S2A,B, Supporting Information). We then found that *Nampt* KD significantly reduced the nuclear localization of NAMPT, and the expression of PD‐L1 was reduced in both the membrane and nucleus. To clarify the impact of NAMPT on the expression of nPD‐L1, we calculated Mandela's colocalization coefficients for PD‐L1 and DAPI; this value decreased from ≈0.36 to 0.12 after *Nampt* KD. We also discovered that IFNγ not only dramatically increased the expression of PD‐L1 and NAMPT, but also enhanced their nuclear localization. After *Nampt* KD, IFNγ stimulation similarly resulted in a significant decrease in the expression of NAMPT, while PD‐L1 mainly manifests as a decrease in nuclear expression levels. In this case, the value of Mandela's colocalization coefficients for PD‐L1 and DAPI decreased from ≈0.41 to 0.20 (Figure 4D,E). Overall, these findings demonstrated that NAMPT enhances the expression of nPD‐L1 in CC cells.

### 2.5 NAMPT Activates SIRT1 to Promote PD‐L1 Gene Expression

**Figure 5:**
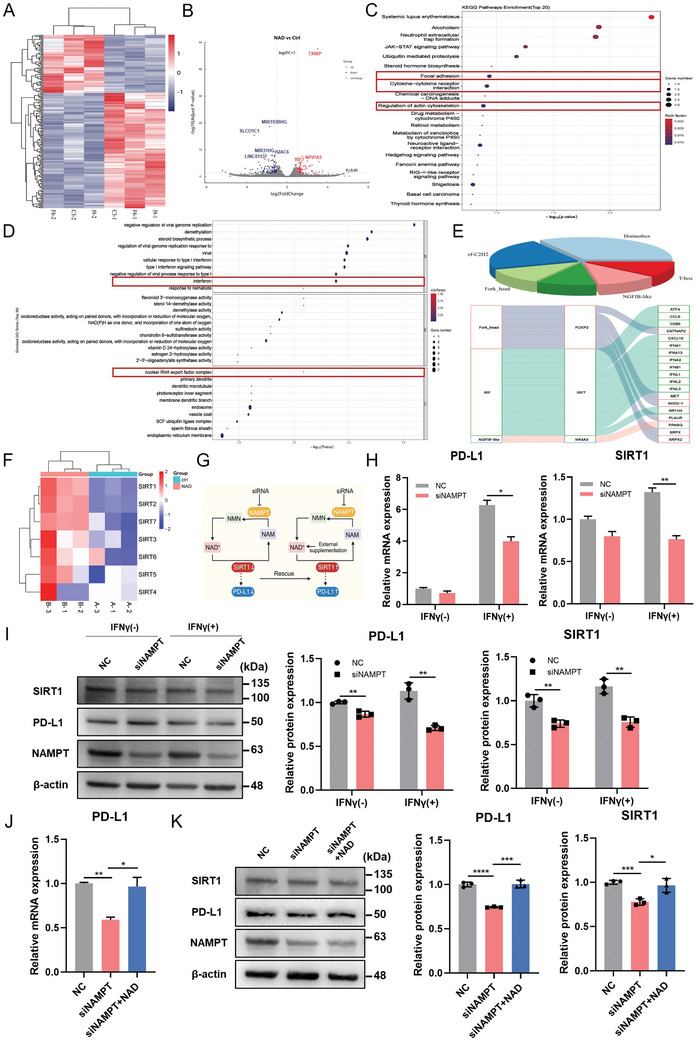
NAMPT activates SIRT1 to promote PD‐L1 gene expression. A) The RNA‐seq heatmap shows the 203 DEGs in HeLa cells treated with exogenous NAD^+^ (1mm) or control (n = 3). B) Volcano plot of DEGs from RNA‐seq showing 68 upregulated genes (red dots) and 135 downregulated (blue dots) genes in HeLa cells treated with exogenous NAD^+^ or control. The fold change is ≥2. C) Top 20 signaling pathways enriched of downregulated DEGs following added with NAD^+^ based on KEGG enrichment analysis. D) Top 30 signaling pathways enriched of upregulated DEGs following added with NAD^+^ based on GO enrichment analysis. E) Annotations of DEGs‐related transcription factors, transcription factor families, and possible target genes. F) The RNA‐seq heatmap illustrating the expression of SIRT1‐7 in HeLa cells after being treated with NAD^+^ or control. G) Schematic diagram of NAD^+^ metabolism–mediated SIRT1 activation to regulate PD‐L1 expression. H, I) The mRNA and protein levels of PD‐L1 and SIRT1 with Nampt KD pretreatment upon or without IFNγ stimulation. J, K) The mRNA and protein levels of PD‐L1 and SIRT1 with NAD^+^ supplementation after Nampt KD. Data were represented as mean ± SD. Two‐tailed unpaired Student's t‐tests. ^*^ *p* < 0.05, ^**^ *p *< 0.01, ^***^ *p* < 0.001, ^****^ *p* < 0.0001.

To further dissect the molecular mechanisms underlying NAD^+^‐dependent regulation of PD‐L1, we performed RNA sequencing (RNA‐seq) on HeLa cells treated with exogenous NAD^+^ (**Figure** **5**A). Our analysis identified 203 differentially expressed genes (DEGs) with statistically significant (*p* < 0.05 and |Log_2_FC| > 1), including 68 upregulated and 135 downregulated genes, as depicted in the volcano plot in (Figure 5B). Kyoto Encyclopedia of Genes and Genomes (KEGG) enrichment analyses were conducted to functionally classify the downregulated DEGs. The results indicated a significant enrichment in pathways related to focal adhesion, cytokine‐cytokine receptor interactions, and regulation of the actin cytoskeleton (Figure 5C). In addition, Gene Ontology (GO) enrichment analyses using upregulated DEGs revealed significant enrichment in pathways related to interferons and the nuclear RNA export factor complex (Figure 5D). Moreover, DEG annotation for identifying transcription factors revealed that they belonged to a variety of families, such as forkhead, interferon regulatory factor, and NGFIB‐like families. We further correlated each transcription factor family with actual transcription factors and performed a predictive analysis of target genes, revealing that genes such as *ATF4, CD80, CXCL10*, and *MET* may serve as target genes (Figure 5E); these genes are closely related to nuclear translocation and immune signaling.^[^ ^46^, ^47^, ^48^, ^49^ ^]^

PTMs can regulate the subcellular localization of proteins, and several studies have reported that histone deacetylases (HDACs) play a crucial role in the transcriptional regulation of cells by deacetylating nuclear histone proteins.^[^ ^50^, ^51^ ^]^The acetylation of PD‐L1 prevents its translocation to the nucleus, leading to a reduction in its nuclear portion.^[^ ^11^ ^]^ Given that sirtuins belong to an evolutionarily conserved NAD^+^‐dependent deacetylase family,^[^ ^52^ ^]^ we explored whether these deacetylases further regulate the transcription of PD‐L1 induced by NAD^+^ metabolism. The analysis of RNA‐seq data from HeLa cells treated with NAD^+^ revealed that *Sirt1* was significantly upregulated after NAD^+^ treatment (Figure 5F).

Several studies have shown that SIRT1 may negatively regulate the expression of inducible PD‐L1; However, it is unclear whether this is directly due to the activity of SIRT1 or indirectly due to NAD^+^ consumption.

However, the enhancement of the NAD^+^‐dependent SIRT1 activity, by adding NMN, partially reversed the PD‐L1 reduction caused by *Sirt1* overexpression.^[^ ^13^ ^]^ However, the regulatory effect of SIRT1 on PD‐L1 expression remains unclear. To investigate how NAMPT regulates SIRT1 and affects PD‐L1 expression, we knocked down *Nampt* and found a decrease in SIRT1 and PD‐L1 expression at both the mRNA and protein levels (Figure 5G–I). However, after *Nampt* KD, supplementation with the direct substrate NMN of SIRT1 significantly reversed the expression of SIRT1 and PD‐L1 at both levels (Figure 5J,K). Taken together, these data suggest that NAMPT selectively activates SIRT1 to promote PD‐L1 transcription.

### 2.6 SIRT1 Inhibitor EX527 Inhibits the Expression and Nuclear Localization of PD‐L1 In Vivo

**Figure 6:**
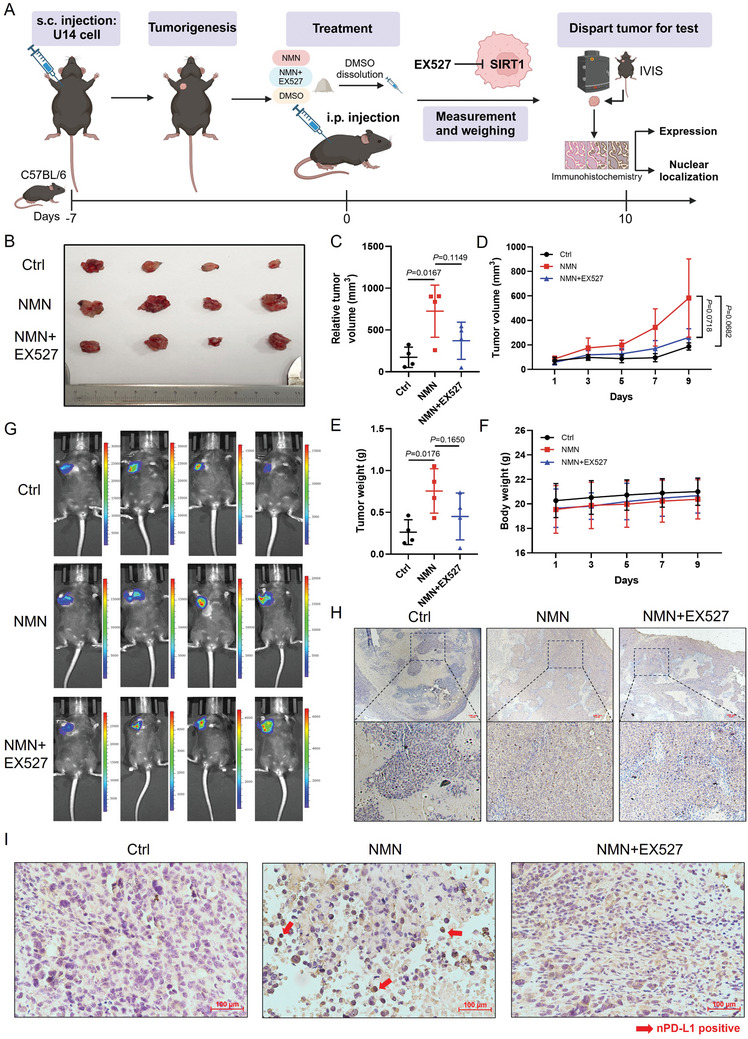
SIRT1 inhibitor EX527 inhibits the expression and nuclear localization of PD‐L1 in vivo. A) Scheme representing the experimental procedure. B–F) Tumor weight (C), relative tumor volume (D), tumor growth curves (E) and body weight (F) of C57BL/6 mice injected subcutaneously with U14 cells with NMN (300mg kg^−1^) alone or NMN combined with SIRT1 inhibitor EX527 (20mg kg^−1^) treatment or solvent control (n = 4). Data are presented as means ± SD. Two‐tailed unpaired Student's t‐tests. G) IVIS images of mice treated with NMN, NMN combined with EX527 or solvent at day 10 post‐injection. H) Representative IHC staining of PD‐L1 expression of tumor tissues in mice treated with NMN, NMN combined with EX527 or solvent. Scale bars, 100 µm. I) Representative IHC staining of nPD‐L1 expression of tumor tissues in mice treated with NMN, NMN combined with EX527 or solvent. Scale bars, 100 µm.

In vivo, experiments were conducted using a subcutaneous U14‐luc tumor model in C57BL/6 mice to examine the effects of SIRT1 on PD‐L1 expression and nuclear localization. Mice were treated with NMN, an NAD^+^ precursor that activates SIRT1 in the presence or absence of EX527, a SIRT1 inhibitor (**Figure** **6**A). The results showed that tumor growth was significantly enhanced by NMN, which is consistent with the role of SIRT1 in promoting tumor progression.^[^ ^53^, ^54^ ^]^ In contrast, cotreatment with EX527 inhibited tumor growth, compared to that with NMN alone, demonstrating that SIRT1 activity accelerated tumor growth and that its inhibition had the opposite effect (Figure 6B–E). In addition, there were no significant differences in body weight among the three groups of mice (Figure 6F). The results were also examined by measuring bioluminescence using an IVIS imaging system (Figure 6G). We also investigated PD‐L1 protein levels in the tumor tissue; IHC staining results showed that the NMN treatment significantly increased the expression of PD‐L1 compared to that in the solvent control group. However, EX527 partially reversed the PD‐L1 upregulation (Figure 6H). Subsequently, we investigated the subcellular localization of PD‐L1. The number of nPD‐L1 positive cells significantly increased in the tumors of mice treated with NMN alone, whereas cotreatment with EX527 effectively suppressed this effect (Figure 6I). These findings align with those of previous reports showing that SIRT1 promotes the deacetylation of nuclear proteins involved in immune escape, including PD‐L1.^[^ ^55^, ^56^ ^]^ However, while the role of SIRT1 in PD‐L1 deacetylation remains consistent with in vivo results, this study uniquely highlights its effect on nuclear localization, which has not been thoroughly explored.

### 2.7 SIRT1‐Mediated Deacetylation and Its Impact on PD‐L1 Nuclear Localization

**Figure 7:**
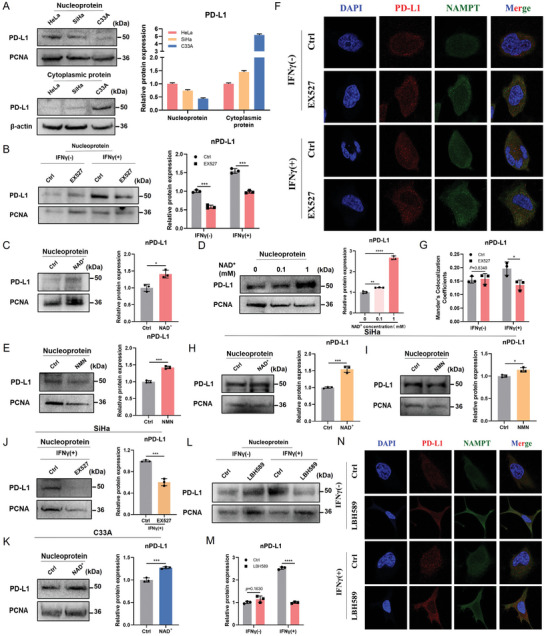
SIRT1 enhances the PD‐L1 nuclear localization. A) Western blot analysis of nuclear and plasma proteins of PD‐L1 in HeLa, SiHa, and C33A cell lines. The right shows the relative quantification of proteins. B) Western blot analysis of nuclear fraction of PD‐L1 in HeLa after treated with SIRT1 inhibitor (EX527) (1 µm). The right shows the relative quantification of proteins. C, D) Western blot analysis of a nuclear fraction of PD‐L1 in HeLa after being treated with different concentrations of NAD^+^ (0.1–1 mm). The right shows the relative quantification of proteins. E) Western blot analysis of a nuclear fraction of PD‐L1 in HeLa after being treated with NMN. The right shows the relative quantification of proteins. F, G) IF staining of a nuclear fraction of PD‐L1 in HeLa after being treated with EX527. (G) shows the Mander's colocalization coefficients of PD‐L1 and DAPI in three random fields of view. Scale bars, 50µm. H, I) Western blot analysis of a nuclear fraction of PD‐L1 in SiHa after being treated with NAD^+^ or NMN. The right shows the relative quantification of proteins. J) Western blot analysis of a nuclear fraction of PD‐L1 in SiHa after being treated with EX527. The right shows the relative quantification of proteins. K) Western blot analysis of a nuclear fraction of PD‐L1 in C33A after being treated with NAD^+^. The right shows the relative quantification of proteins. L, M) Western blot analysis of a nuclear fraction of PD‐L1 in HeLa after being treated with an HDAC inhibitor (LBH589) (50 nm). (m) shows the relative quantification of proteins. N) IF staining of nuclear fraction of PD‐L1 in HeLa after supplying with LBH589. Scale bars, 50µm. Data are presented as the means ± SD. Two‐tailed unpaired Student's t‐tests. ^*^ *p* < 0.05, ^**^ *p* < 0.01, ^***^ *p* < 0.001, ^****^ *p* < 0.0001.

To further understand the molecular basis of SIRT1 regulation of PD‐L1, we performed in vitro experiments to determine nuclear and plasma PD‐L1 protein levels in HeLa, SiHa, and C33A cell lines. Consistent with the IF results (Figure 4A), PD‐L1 nuclear expression was relatively high in HeLa cells, whereas cytoplasmic PD‐L1 expression was comparatively high in C33A cells (**Figure** **7**A). We first treated HeLa cells with the SIRT1 inhibitor EX527 and observed a significant reduction in nPD‐L1 levels, especially when stimulated by IFNγ (Figure 7B). Correspondingly, we activated SIRT1, by adding NAD^+^ to the medium, and found that the expression of nPD‐L1 significantly increased, by ≈2‐fold (Figure 7C). We further treated HeLa cells with different concentrations of NAD^+^ and found that nPD‐L1 levels increased in an NAD^+^ concentration‐dependent manner (Figure 7D). In addition, NMN treatment significantly increased nPD‐L1 levels (Figure 7E). We further validated the effect of SIRT1 on PD‐L1 nuclear localization using IF staining. The results showed that SIRT1 inhibition led to a slight increase in *Nampt* expression; however, this effect was not significant when stimulated with IFNγ. Moreover, compared to the control, no significant changes in the expression of nPD‐L1 were observed in the group treated with EX527 alone. However, upon IFNγ stimulation, the inhibitory effect of EX527 on nPD‐L1 expression was enhanced. Notably, Mandela's colocalization coefficient value for PD‐L1 and DAPI decreased from ≈0.20 to 0.14, which is consistent with the WB result (Figure 7F,G). We also validated this result in the SiHa and C33A cell lines. We found that, regardless of the basal levels, NAD^+^ or NMN treatment significantly increased nPD‐L1 levels in such cell lines, whereas inhibition of SIRT1 led to a decrease in nPD‐L1 levels in SiHa cells, which is consistent with the phenomenon observed in HeLa cells (Figure 7H–K).

To demonstrate that SIRT1 enhances the nuclear localization of PD‐L1 through deacetylation, we used HDACs as a positive control and treated HeLa cells with the HDAC inhibitor panobinostat (LBH589).^[^ ^57^, ^58^ ^]^ The results revealed that LBH589 alone had no apparent effect on the expression of nPD‐L1, but, when stimulated with IFNγ, LBH589 had a significant inhibitory effect on the expression of nPD‐L1, which is consistent with the results of the EX527 treatment (Figure 7L,M). This result was validated using IF staining. In addition, LBH589 treatment resulted in significant changes in cell morphology, and the nuclear localization of PD‐L1 was remarkably inhibited (Figure 7N). These results suggest that SIRT1 enhances the expression of nPD‐L1 through deacetylation.

**Figure 8:**
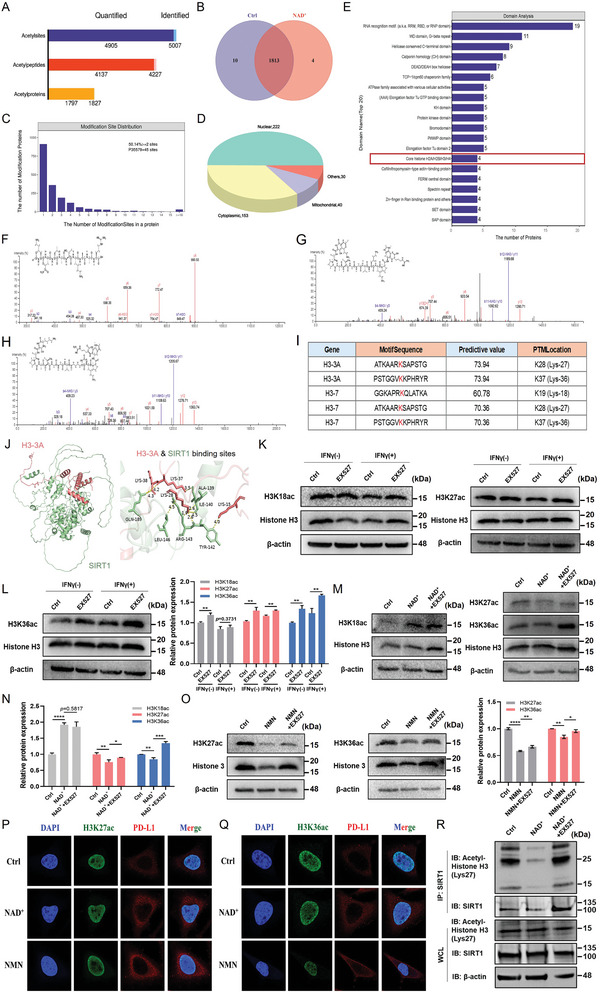
SIRT1‐mediated deacetylation and its impact on PD‐L1 nuclear localization. A) The number of acetylated proteins, peptides, and sites identified and quantifiable in acetyl‐proteomics. B) Venn diagram showing the overlap of protein numbers in NAD^+^‐treated and control groups. C) Histogram showing the distributions of the number of acetylation sites per protein. D) The subcellular distribution of proteins to which differentially expressed modified peptides belong. E) Bar chart showing the number of proteins to which modified peptides belong in the domain. F–H) Interacting proteins analyzed by LC‐MS/MS assay. The peptide spectrum of Histone H3K18ac (F), H3K27ac (G), and H3K36ac (H) were identified and the N‐terminal and C‐terminal collision‐induced dissociation fragment ions were indicated by b and y, respectively. I) List of predicted lysine acetylation sequences and sites by mass spectrum. The predictive value of motif sequence results in greater than 13 are generally considered reliable. J) Molecular docking (left) and binding site prediction (right) of H3‐3A and SIRT1. K, L) Western blot analysis of H3K18ac, H3K27ac, and H3K36ac after being treated with EX527 upon or without IFNγ stimulation. (L) shows the relative quantification of proteins. M, N) Western blot analysis of H3K18ac, H3K27ac, and H3K36ac after being treated with NAD^+^, NAD^+^ combined with EX527 or control. (N) shows the relative quantification of proteins. O) Western blot analysis of H3K27ac and H3K36ac after being treated with NMN, NMN combined with EX527, or control. The right shows the relative quantification of proteins. P, Q) IF staining of PD‐L1, H3K27ac, and H3K36ac after treated with NAD^+^ or NMN. Scale bars, 50 µm. R) IB analysis of WCL and anti‐SIRT1 IPs derived from HeLa cells treated with NAD^+^, NAD^+^ combined with EX527, or control. Data are presented as the means ± SD. Two‐tailed unpaired Student's t‐tests. ^*^ *p* < 0.05, ^**^ *p *< 0.01, ^***^ *p* < 0.001, ^****^ *p* < 0.0001.

To systematically describe the deacetylation mediated by SIRT1, we conducted an acetyl proteomic analysis based on affinity‐directed mass spectrometry (MS). Lysates from HeLa cells, with or without NAD^+^ treatment, were subjected to tryptic digestion, acetylated peptide enrichment, and LC‐MS/MS precursor signal intensity analysis. A total of 5007 acetyl‐sites and 4227 acetylpeptides matching 1827 acetyl proteins were identified; 1813 of these acetyl‐proteins in both the control and NAD^+^ treated groups (**Figure** **8**A,B). A quantitative analysis was conducted on all identified acetylation sites to analyze the distribution of modification sites on the proteins. The results showed that 50.14% of the proteins had two or more modification sites (Figure 8C). We further screened for significantly differentially modified peptides and found that 259 peptides were upregulated and 218 were downregulated in the NAD^+^ group compared to the control group. Subcellular localization analyses of proteins belonging to the differentially modified peptides revealed that approximately half of the proteins were located in the nucleus (Figure 8D). Furthermore, the analysis of the structural domains of these proteins, showed that they were enriched in domains such as those of the ATPase family associated with various cellular activity and core histones H2A/H2B/H3/H4 (Figure 8E).

Histone H3 acetylation is closely related to the expression of PD‐L1 in multiple cancers.^[^ ^59^, ^60^, ^61^ ^]^ To clarify the acetylation site of histone H3, we analyzed the identified modified peptides and discovered that SIRT1‐mediated deacetylation may act on H3K18, H3K27, and H3K36 (Figure 8F–I). Moreover, we performed molecular docking of SIRT1 and histone H3 using PyMol 2.4. Consistently, the analysis suggested that histone H3 primarily binds to the corresponding sites of SIRT1 through Lys‐27 and Lys‐36 (Figure 8J), suggesting that histone H3K27 and H3K36 may be targets for SIRT1‐mediated deacetylation.

Importantly, we verified whether SIRT1 affects H3K18, H3K27, and H3K36 acetylation in HeLa cells. After treatment with EX527, the acetylation levels of H3K27 and H3K36 increased with or without IFNγ stimulation. However, the acetylation of H3K18 only increased when cells were treated with EX527 alone, and did not show significant changes when stimulated with IFNγ (Figure 8K,L). To investigate whether NAD^+^ metabolism affects H3K18ac, H3K27ac, and H3K36ac through SIRT1, we next treated HeLa cells with the SIRT1 substrate NAD^+^. The results showed that the acetylation level of H3K18 significantly increased after NAD^+^ treatment, indicating that histone H3K18 is not a binding site of SIRT1 related to NAD^+^ metabolism. Notably, the expression of H3K27ac and H3K36ac was reduced after NAD^+^ treatment, whereas pretreatment with EX527 partially attenuated this effect (Figure 8M,N). Additionally, we used NMN, a precursor of NAD^+^, to verify whether H3K27 and H3K36 were SIRT1 binding sites. Similarly, NMN treatment decreased H3K27ac and H3K36ac levels, and this effect was weakened by EX527 pretreatment (Figure 8O). Consistent with previous results, IF analyses on HeLa cells treated with NAD^+^ and NMN to investigate the expression levels of nPD‐L1, H3K18ac, and H3K27ac showed that both NAD^+^ and NMN treatments increased the expression of nPD‐L1and decreased in the expression of H3K27ac and H3K36ac (Figure 8P,Q).

We further analyzed the interactions between SIRT1 and histone H3K27. The results showed that NAD^+^ treatment significantly reduced the physical interaction between SIRT1 and H3K27ac, possibly due to the deacetylation of H3K27ac by SIRT1. However, EX527 pretreatment restored the physical interaction between SIRT1 and H3K27ac (Figure 8R).

In conclusion, our acetyl proteomic analysis and subsequent experiments revealed a novel mechanism through which SIRT1‐mediated deacetylation of histones H3K27 and H3K36 influences PD‐L1 nuclear localization and immune evasion. This finding suggests that the acetylation status of H3K27 may directly or indirectly regulate PD‐L1 nuclear localization, providing a potential therapeutic target for modulating PD‐L1 levels and enhancing immunotherapy efficacy.

### 2.8 Comparison Between SIRT1 and HDACs in Regulating PD‐L1

**Figure 9:**
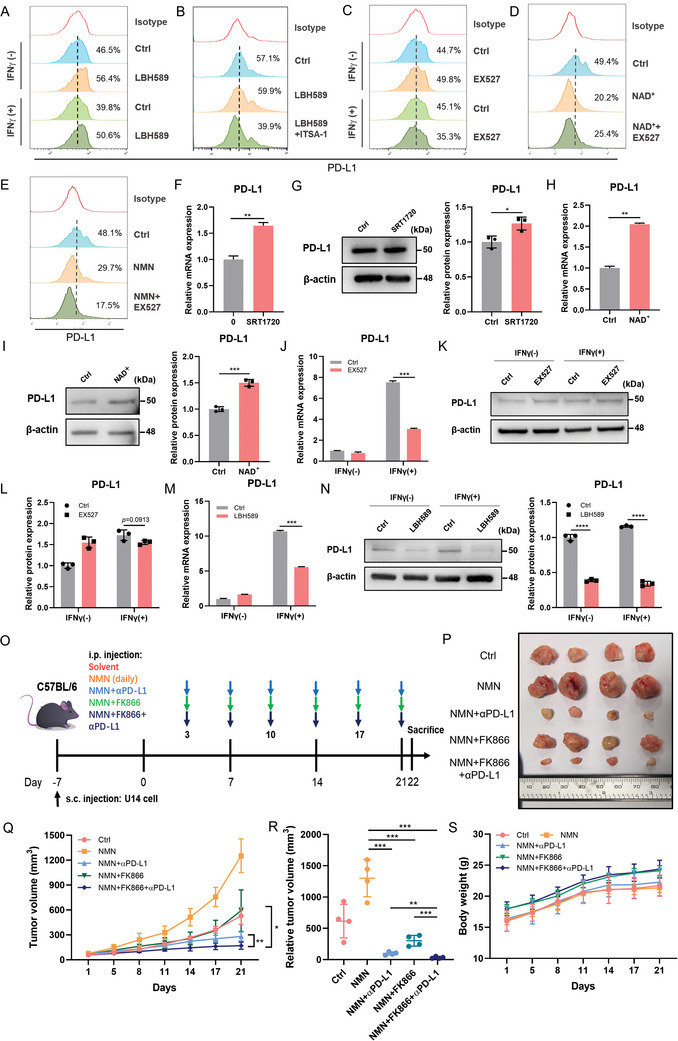
Comparison Between SIRT1 and HDACs in Regulating PD‐L1. A) Flow cytometry of membrane PD‐L1 expression after supplying with LBH589 in HeLa. B) Flow cytometry of membrane PD‐L1 expression after supplying LBH589 alone or in combination with ITSA‐1 (50 µm) pretreatment in HeLa. C) Flow cytometry of membrane PD‐L1 expression after supplying with EX527 in HeLa. D, E) Flow cytometry of membrane PD‐L1 expression after supplying with NAD^+^ or NMN alone or in combination with EX527 pretreatment in HeLa. F, G) qPCR and Western blot analysis of overall expression of PD‐L1 after supplying with SIRT1 activator SRT1720 (160 nm). H, I) qPCR and Western blot analysis of overall expression of PD‐L1 after supplying with SIRT1 substrate NAD^+^. J–L) qPCR and Western blot analysis of overall expression of PD‐L1 after supplying with EX527. M, N) qPCR and Western blot analysis of overall expression of PD‐L1 after supplying with LBH589. O) Scheme representing the experimental procedure. NMN (150mg kg^−1^) was administered daily with water. FK866 (20mg kg^−1^) and αPD‐L1(3mg kg^−1^) are injected intraperitoneally twice a week. P–S) Tumor size (P), tumor growth curves (Q), tumor volume (R), and body weight (S) of C57BL/6 mice injected subcutaneously with U14 cells (n = 4). Data were represented as mean ± SD. Two‐tailed unpaired Student's t‐tests. ^*^ *p* < 0.05, ^**^ *p *< 0.01, ^***^ *p* < 0.001.

HDAC2 has been reported to deacetylate membrane PD‐L1, thereby allowing its transport from the membrane to the nucleus.^[^ ^11^ ^]^ To compare the regulatory effects of SIRT1 and HDACs on membrane PD‐L1, we treated HeLa cells with the HDAC inhibitor LBH589 and found an increase in membrane PD‐L1 expression, whereas this upregulation was significantly inhibited after treatment with the HDAC activator ITSA‐1, demonstrating the deacetylation regulatory effect of HDACs on membrane PD‐L1 (**Figure** **9**A,B). However, SIRT1 inhibition did not enhance the expression of membrane‐bound PD‐L1 (Figure 9C). We further activated SIRT1 using its substrates, NAD^+^ or NMN and observed a significant decrease in membrane PD‐L1 expression. Furthermore, pretreatment with EX527 did not reverse this result (Figure 9D,E), indicating that SIRT1 is not involved in regulating the deacetylation of membrane‐bound PD‐L1.

We also compared the effects of SIRT1 and HDACs on PD‐L1 total protein levels. We first increased the activity of SIRT1 using the specific activator SRT1720 and substrate NAD^+^ and observed an increase in PD‐L1 expression at both the mRNA and protein levels (Figure 9D–G). However, when EX527 was used to inhibit SIRT1, the expression of PD‐L1 was significantly suppressed at the mRNA level, while the total protein level of PD‐L1 did not show significant downregulation (Figure 9H,I), indicating that SIRT1‐mediated deacetylation activity may mainly affect PD‐L1 nuclear localization. We also investigated the effect of LBH589 on PD‐L1 expression. We observed that LBH589 treatment significantly inhibited the transcription of PD‐L1 (Figure 9K), which is consistent with SIRT1 expression. However, at the protein level, LBH589 and EX527 treatments showed completely different effects. LBH589 significantly reduced the expression of PD‐L1 protein both with and without IFNγ stimulation (Figure 9J). In conclusion, SIRT1‐mediated deacetylation mainly affects the nuclear localization of PD‐L1 rather than the membrane and total PD‐L1 protein levels, which is different from the role of HDACs.

### 2.9 Investigating the Efficacy of Targeting NAMPT‐Mediated NAD^+^ Biosynthesis in Anti‐PD‐L1 Immunotherapy

To initially explore the impact of NAMPT‐mediated NAD^+^ metabolism on the efficacy of anti‐PD‐L1 immunotherapy in cervical cancer, we established a subcutaneous tumor model in C57BL/6 mice using U14‐luc cells. A cervical cancer model with high NAD+ metabolism levels was generated by administering NMN (150 mg kg^−1^). We then evaluated the therapeutic efficacy of FK866 and αPD‐L1 alone or in combination (Figure 9O). Throughout the experiment, no significant changes in mouse body weight were observed (Figure 9S).

Importantly, FK866 treatment alone effectively reversed the tumor‐promoting effect of NMN, highlighting the importance of correcting NAD^+^ metabolism disorders in inhibiting cervical cancer progression.

Additionally, while αPD‐L1 monotherapy significantly slowed tumor growth, combination therapy with FK866 and αPD‐L1 achieved optimal tumor inhibition (Figure 9P–R). Specifically, the combination therapy reduced tumor volume by 40.5% compared to αPD‐L1 therapy alone, underscoring the clinical potential of targeting NAD^+^ metabolism to enhance the efficacy of anti‐PD‐L1 immunotherapy in CC.

**Figure 10:**
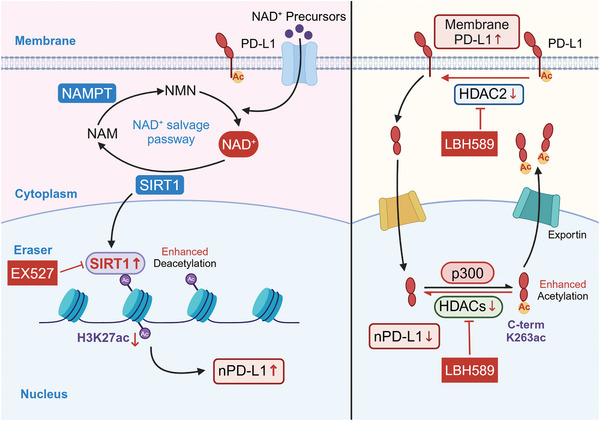
A Schematic summary of NAD^+^ metabolism reprogramming driving SIRT1 inducing PD‐L1 nuclear localization. Left Panel: This schematic illustrates how the reprogramming of NAD^+^ metabolism leads to SIRT1‐dependent deacetylation, which in turn promotes the nuclear localization of PD‐L1. This process is a key mechanism by which the epigenetic landscape within the cell is altered, potentially impacting immune evasion strategies employed by cancer cells. Right Panel: Conversely, this pane is achieved through the deacetylation of membrane‐bound PD‐L1, a process mediated by HDAC2 (according to reference,^[^ ^11^ ^]^ License Number 5915730192999).

This figure encapsulates the dual mechanisms by which PD‐L1 subcellular distribution is controlled, emphasizing that SIRT1 appears to play a more significant role in the regulation of PD‐L1 nuclear localization compared to other deacetylases, underscoring the complexity of immune checkpoint regulation and its implications for cancer therapy resistance(**Figure** **10**).

## 3 Discussion

In summary, our study reveals that targeting the NAD^+^ metabolism enhances the efficacy of anti‐PD‐L1 immunotherapy in CC. FK866, an NAD^+^ biosynthesis inhibitor, reversed the tumor‐promoting effects of NMN, highlighting the importance of correcting NAD^+^ metabolism disorders in inhibiting cervical cancer progression. The combination of FK866 and αPD‐L1 achieved significant tumor inhibition. These results, which are consistent with previously reported research, further emphasize the clinical potential of targeting NAD^+^ metabolism to augment the efficacy of anti‐PD‐L1 immunotherapy across diverse cancer types. Lv et al.^[^ ^13^ ^]^ demonstrated that NAMPT drives PD‐L1 expression across various cancer types, highlighting its role in immune evasion. Similarly, Li et al.^[^ ^62^ ^]^ showed that NAMPT inhibition enhances the efficacy of PD‐L1 blockade in murine brain tumor glioblastoma.

Mechanistically, PD‐L1 overexpression is regulated by both transcriptional and post‐transcriptional processes, with SIRT1 playing a central role in histone H3 deacetylation, influencing PD‐L1 subcellular distribution. NAMPT‐mediated NAD^+^ biosynthesis induces H3K27 deacetylation, promoting tumor immune evasion^[^ ^61^, ^63^, ^64^ ^]^ This discovery unveils a metabolic‐epigenetic pathway governing PD‐L1 regulation and proposes potential therapeutic interventions to enhance the efficacy of immunotherapy in CC.

Consistent with recent studies highlighting the role of histone acetylation, particularly H3K27ac in regulating gene expression and immune responses in cancer,^[^ ^65^, ^66^, ^67^ ^]^ our findings suggest an association between the acetylation status of H3K27 and PD‐L1 expression in multiple cancers. The enhanced interaction between H3K27ac and PD‐L1 following NAD^+^ treatment suggests that PD‐L1 nuclear localization and function are regulated by histone acetylation. This is further supported by the observation that nPD‐L1 is associated with immune evasion in tumors,^[^ ^10^, ^68^, ^69^ ^]^ indicating that H3K27 acetylation may regulate PD‐L1 nuclear localization and contribute to immune escape mechanisms. Our findings have significant therapeutic implications. We showed that the pharmacological inhibition of SIRT1 using EX527 reduced tumor growth in vivo and lowered nuclear PD‐L1 levels, thereby enhancing the antitumor immune response. Conversely, NMN, an NAD^+^ precursor that activates SIRT1,^[^ ^70^, ^71^ ^]^ increased tumor growth and PD‐L1 expression. This reinforces the concept that the NAD^+^/SIRT1 axis is a pivotal regulator of immune escape in CC, suggesting the potential of combination therapies targeting this axis.

In summary, our study revealed a novel regulatory mechanism for the nuclear translocation of PD‐L1 in CC; this translocation is mediated by SIRT1, promoting immune escape through histone H3 deacetylation. We demonstrated that SIRT1 inhibition by EX527 reduced PD‐L1 nuclear localization, enhanced the antitumor immune response, and significantly diminished tumor growth in vivo. Our findings offer a new perspective on the role of nPD‐L1 in immune evasion and suggest that this nuclear localization may contribute to the resistance to PD‐1/PD‐L1 immune checkpoint blockade therapies.

Considering the clinical applications, our study aligns with previous reports indicating that histone deacetylase inhibitors (HDACis) can sensitize tumors to immune checkpoint inhibition.^[^ ^72^, ^73^, ^74^ ^]^

Our work provides mechanistic support for this strategy by showing that SIRT1‐mediated histone deacetylation promotes the accumulation of nPD‐L1, highlighting the therapeutic potential of combining an anti‐PD‐1/PD‐L1 therapy with HDACis or SIRT1 inhibitors to reduce nPD‐L1expression and improve treatment efficacy in CC.

Our study suggests that epigenetic regulation plays a crucial role in the modulation of immune checkpoints. Histone modifications, such as deacetylation, have been implicated in altering chromatin structure and gene transcription, influencing the expression of key regulators of immune evasion, such as PD‐L1.^[^ ^75^, ^76^, ^77^ ^]^ Notably, after translocation to the nucleus, PD‐L1 binds DNA, promotes the expression of immune response genes (such as *RelB, TRAF1, HLAs*), and facilitates the inflammatory response. In addition, PD‐L1 upregulates the expression of other immune checkpoint genes, such as PD‐L2 and VISTA, which in turn makes tumor cells resistant to PD1/PD‐L1 blockade therapy.^[^ ^11^ ^]^

The specificity of SIRT1 in deacetylating histones H3K27 and H3K36 adds another layer of complexity to PD‐L1 regulation, as previous studies have linked these histone marks to the repression of immune genes r.^[^ ^78^, ^79^, ^80^ ^]^ By identifying these modifications, we provide a more detailed understanding of how SIRT1 influences immune checkpoint regulation at the epigenetic level.

Our findings highlight the critical role of the NAD^+^/SIRT1/PD‐L1 axis in promoting immune escape in CC via epigenetic modifications. By demonstrating that SIRT1‐mediated deacetylation of histone H3 promotes PD‐L1 nuclear localization, we uncovered a novel mechanism by which cancer cells evade immune surveillance. These insights offer new opportunities for therapeutic interventions, particularly to enhance the efficacy of immune checkpoint inhibitors in CC. Targeting SIRT1, either alone or in combination with NAD^+^ metabolism modulators, holds promise as a novel strategy for overcoming immune resistance in CC.

## 4 Limitations

Our study has several limitations. First, although we observed significant effects of SIRT1 inhibition on nPD‐L1 nuclear localization, the functional role of nuclear PD‐L1 in immune evasion remains unclear. Future studies should focus on characterizing the transcriptional targets of nuclear nPD‐L1 and their contribution to immune escape. Moreover, the precise interactions between NAD^+^ metabolism and other HDACs involved in PD‐L1 regulation warrant further investigation. Finally, although we established a link between SIRT1 activity and nPD‐L1, the precise association between nPD‐L1 and specific genetic backgrounds in patients with CC remains unclear. Previous studies have shown that nPD‐L1 can occur in different cancer types, including lung and colon cancers, which are not associated with the classic mutations commonly observed in uveal melanoma. Future research should explore the genetic factors that promote nPD‐L1 signaling to better understand its role in diverse cancer contexts.

## 5 Experimental Section

### Patient and Ethics Approval

CC, CIN, and gallbladder cancer tissues were collected from the Affiliated Women's Hospital of Jiangnan University for IF staining (**Table** **1**). Clinical samples were used only with patient consent and the approval of the institutional research ethics committee. (approval No.2020‐01‐0309‐06)

**Table 1:** Clinical characteristics of CC, CIN, and gallbladder cancer patients.

Patient No.	Age [yr]	Tumor type	FIGO stage	Drug‐resistant
1	55	Cervical adenocarcinoma	IIb	No
2	39	Cervical squamous cell carcinoma	IIa2	Yes
3	63	Cervical squamous cell carcinoma	IIIC2r	Yes
4	77	Cervical squamous cell carcinoma	IIIC1r	Yes
5	65	Cervical squamous cell carcinoma	IVA	Yes
6	53	Cervical adenocarcinoma	IIIC1(p)	Yes
7	33	Cervical squamous cell carcinoma	IB1	No
8	61	Cervical squamous cell carcinoma	IIIC1p	Yes
9	47	Cervical adenocarcinoma	IIA2	Yes
10	58	Cervical squamous cell carcinoma	IIIC1(p)	Yes
11	44	Cervical squamous cell carcinoma	IIa2	Yes
12	36	Cervical adenocarcinoma	IB2	Yes
13	65	Cervical squamous cell carcinoma	IIIB	Yes
14	58	Cervical squamous cell carcinoma	IIIC1	Yes
15	55	Cervical squamous cell carcinoma	Ib2	Yes
16	50	Cervical squamous cell carcinoma	IVB	Yes
17	52	Cervical squamous cell carcinoma	IIIB	Yes
18	72	Gastric‐type endocervical adenocarcinoma	IIIC1p	Yes
19	39	Cervical squamous cell carcinoma	IB1	Yes
20	71	Cervical squamous cell carcinoma	IIIC1p	No
21	58	Cervical adenocarcinoma	IIIC2P	No
22	50	Cervical squamous cell carcinoma	IVB	Yes
23	58	Cervical squamous cell carcinoma	IIIC1r	No
24	53	Cervical squamous cell carcinoma	IIIC1(p)	No
25	34	Cervical squamous cell carcinoma	IB2	No
26	38	Cervical squamous cell carcinoma	IIIC1(r)	No
27	63	Gallbladder cancer	IVB	No
28	39	Cervical intraepithelial neoplasia	II‐III	/
29	30	Cervical intraepithelial neoplasia	II‐III	/
30	52	Cervical intraepithelial neoplasia	III	/

### Animal Models

All mice were maintained in a pathogen‐free environment, subjected to a diurnal photoperiod of 12 h of light and 12 h of darkness, and thermoregulated at a temperature range of 20–25 °C. They were provided with ad libitum access to standard rodent chow (Product Standard No. Q031/0120000099C001‐2015, Shanghai Shilin Biologic Science & Technology, China) and water. The experiments comprised exclusively male mice, with an average age of 6 weeks at the commencement of the study. The health status of the mice was monitored in accordance with established protocols, and all animal experimental protocols were reviewed and approved by the Institutional Animal Care and Use Committee (IACUC) at Jiangnan University. For in vivo studies, 1×10^6^ U14‐luc cells were injected subcutaneously into the right forelimbs of C57BL/6 mice (Laboratory Animal Resources, Chinese Academy of Sciences, Shanghai, China). The number of mice per group for each experiment is detailed in the figure legends. Tumor size (length × width^2^× 0.5) was measured once every 2 days after injection. The mice were euthanized and the tumors were harvested for weight measurement and further tissue analyses.

### In Vivo Imaging

After subcutaneous inoculation of U14‐luc cells, before sacrifice, fluorescent enzyme substrate D‐Luciferin was intraperitoneally injected at a dose of 150 mg kg^−1^. The surgery procedure began 5–10 min after injection. The mouse was dissected and imaged for ICG and Luc signals using the IVIS Lumina Series III live imaging system. The intestine was then collected and the ICG and Luc signals of the disseminated tumor nodules and adjacent intestine were analyzed.

### Chemicals and Reagents

NAD^+^ (B27081) and NAM (B21424) were purchased from Shanghai Yuanye Bio‐Technology. NMN (N131850) and FK866 (F425320) were purchased from Aladdin. EX527 (S1541) and LBH589 (S1030) were purchased from Selleck. IFNγ (Z02986) was purchased from Genscript. SRT1720 (HY‐10532), ITSA‐1 (HY‐100508), and αPD‐L1 (HY‐P99145) were purchased from MCE. 

### Cell Culture

HeLa (cervical adenocarcinoma) and SiHa (squamous carcinoma of the cervix) cell lines were purchased from the Cell Bank of Type Culture Collection of the Chinese Academy of Sciences (CBTCCCAS). C33A (human cervical cancer cells) and U14 (mouse cervical cancer cells) were purchased from Procell. HeLa and U14 were grown in RPMI 1640 Medium (HyClone, USA), SiHa was grown in Dulbecco's Modified Eagle's Medium (DMEM, HyClone, USA), C33A was grown in MEM liquid culture Medium (Boster). All cell lines were supplemented with 10% fetal bovine serum (VivaCell) at 37 °C and 5% CO_2_.

### Small Interfering RNA Molecules

siRNA targeting human Nampt was purchased from Biotend (Shanghai, China). The target sequences were listed in **Table** **2**. siRNA was transfected into cells using RFect (BAIDAI) following the manufacturer's instructions for 48h.

**Table 2:** Primers sequences for siRNA.

Gene	Forward (5′ to 3′)	Reverse (5′ to 3′)
si‐NAMPT	GAAGCCAAAGAUGUCUACAAATT	UUUGUAGACAUCUUUGGCUUCTT
negative control	UUCUCCGAACGUGUCACGUTT	ACGUGACACGUUCGGAGAATT

### RNA Extraction and qRT‐PCR

Total RNA was extracted from cells by using RNA‐easy Isolation Reagent (Vazyme) and reverse transcribed by PrimeScript RT reagent kit (Vazyme). The RT‐qPCR was performed using SYBR Green Master Mix (Vazyme) by an ABI ViiA7500 Real‐Time System (Life Technologies). The primers (**Table** **3**) were designed by the NCBI primer tools.

**Table 3:** Primers sequences for RT‐PCR.

Gene	Species	Forward (5′ to 3′)	Reverse (5′ to 3′)
NAMPT	Human	TAAAAGCTGTTCCTGAGGGCT	AGAATTTGTGGCCACTGTGATTG
NAPRT	Human	GTGAGGTGAATGTCATTGG	GGCCACCAGCTTATAGAC
PD‐L1	Human	TGCCGACTACAAGCGAATTACTG	CTGCTTGTCCAGATGACTTCGG
SIRT1	Human	TGACCTCCTCATTGTTATTGGG	GGCATACTCGCCACCTAACCT
ACTB	Human	TCCATCATGAAGTGTGACGT	TACTCCTGCTTGCTGATCCAC

### Western Blotting and Antibodies

Cellular lysates were obtained using the Efficient RIPA tissue/cell rapid lysis solution (Solarbio) on an orbital shaker at a temperature of 4 °C for a duration of 20 min. Following this, the lysates were subjected to centrifugation at 12000×g at 4 °C for an additional period of 20 min to sediment the cellular debris. The resulting protein‐containing supernatants were then quantified employing the BCA protein concentration determination kit (Beyotime). Before electrophoresis, the protein samples were denatured by boiling with an SDS loading buffer for a period of 15 min. The proteins were separated by 8% or 10% (wt/vol) SDS‐PAGE (Boster) and transferred to PVDF (Beyotime) membranes. The membranes were blocked with Protein free rapid sealing solution (Boster) for 15 min and incubated with the primary antibodies at 4 °C overnight and the corresponding secondary antibodies for 2 h at room temperature. Finally, the band signals were visualized by BIO‐RAD ChemiDoc XRS+. The following primary antibodies were used in this study: anti‐NAMPT (11776‐1‐AP), anti‐NAPRT (66159‐1‐1g), anti‐PD‐L1 (66248‐1‐lg), anti‐PCNA (60097‐1‐lg) were purchased from Proteintech. Anti‐SIRT1 (BM3929), anti‐Histone H3 (A12477‐2) and anti‐β‐actin (BA2305) were purchased from Boster. Anti‐Acetyl‐Histone H3 (Lys18) (PTM‐114RM), anti‐Acetyl‐Histone H3 (Lys27) (PTM‐116RM) and anti‐Acetyl‐Histone H3 (Lys36) (PTM‐117RM) were purchased from PTM BIO.

### Nuclear Fractionation

The cellular nuclear and cytosolic fractions were isolated using a nuclear and cytoplasmic protein extraction kit (Beyotime, P0028) according to the manufacturer's instructions. Briefly, a 20 µL aliquot of the cellular pellet was resuspended in 200 µL of cytoplasmic protein extraction buffer A and placed on ice for 15 min. Next, 10 µL of cytoplasmic protein extraction buffer B was added, and the mixture was incubated for an additional minute. The sample was then centrifuged at 12000×g for 5 min to separate the cytoplasmic and nuclear fractions. The supernatant, enriched for cytoplasmic proteins, was collected, while the pellet was re‐suspended in 50 µL of nuclear protein extraction buffer to extract nuclear proteins. The resulting protein fractions were subsequently prepared for western blot analysis.

### RNA‐seq

To perform RNA‐seq, total RNA was isolated from the cultured cells by TRIzol reagent (Invitrogen, Carlsbad, CA, USA). The integrity of the RNA was confirmed by a 2100 Bioanalyzer (Agilent Technologies, USA), and the concentration was measured by a Qubit 2.0 fluorometer with a Qubit RNA assay kit (Life Technologies, Carlsbad, CA, USA). Then, sequencing libraries were generated using an Illumina TruSeq RNA Sample Prep Kit (San Diego, CA, USA). The libraries were finally sequenced on the Illumina HiSeq 2500 platform.

### Immunoblot and Immunoprecipitation

For detection of SIRT1 and H3K27ac interaction, HeLa cells were either untreated or treated with NAD^+^ or NAD^+^ combined with EX527. Cell extracts were prepared in radioimmune precipitation assay buffer (1 PBS, 1% Nonidet P‐40, 0.1% SDS, 100 g mL^−1^ phenylmethylsulfonyl fluoride, aprotinin 1 g mL^−1^), and lysates were incubated with anti‐SIRT1 antibody or control rabbit serum and antibody complexes was isolated using protein G‐agarose beads (Amersham Biosciences) and washed three times with radioimmune precipitation assay buffer. The beads were then boiled in an SDS sample buffer and run on a 10% SDS PAGE. Samples were transferred to nitrocellulose filters and subjected to Western blotting using an anti‐acetyl‐histone H3 (Lys27) antibody.

### Immunofluorescence Staining

Deparaffinized and rehydrated tissue samples or cells seeded on glass slides were fixed with 4% formaldehyde for 30 min, blocked with Protein rapid sealing solution (Boster) for 1 h, and permeabilized with 0.5% Triton X‐100 for 15 min. Then, they were incubated with the primary antibodies for 2h and the appropriate fluorescent secondary antibodies for 1 h at room temperature. The nuclei were stained with Anti fluorescence quenching sealing solution (including DAPI) (Beyotime). Finally, IF images were taken with a Nikon ECLIPSE Ti2 inverted microscope.

### Immunohistochemistry

IHC staining was performed on representative tissue sections from formalin‐fixed and paraffin‐embedded tissue blocks from human CC and mice tumor xenografts using the following antibodies at the indicated concentrations: anti‐PD‐L1 (Proteintech).

### Flow Cytometry Analysis

Single‐cell suspensions were prepared from cells in culture. Cells were incubated with Purified Anti‐Human CD16 Antibody (E‐AB‐F1236A, Elabscience) for 30min. Then they were incubated with the primary antibodies or isotype (66360‐1‐1g, Proteintech) for 2h and the appropriate fluorescent secondary antibodies for 1 h at room temperature. Resuspend the cells in 50 µL of Flow Cytometry Staining Buffer (Elabscience). Data were acquired immediately by a BriCyte E6 flow cytometer and analyzed using FlowJo software.

### High‐Performance Liquid Chromatography (HPLC) and LC‐MS/MS Analysis

Each protein fraction was subjected to nanoLC‐MS/MS for analytical profiling. The mobile phases were defined as follows: Mobile phase A, consisting of 0.1% formic acid in water, and Mobile phase B, a solution of 84% acetonitrile supplemented with 0.1% formic acid. The chromatographic system was initialized with a 95% composition of solvent A. The peptide samples were introduced onto a reverse‐phase trap column (Thermo Scientific Acclaim PepMap 100, 100 µm × 2 cm, nanoViper C18) interfaced with a C18 reversed‐phase analytical column (Thermo Scientific Easy Column, 10 cm long, 75 µm inner diameter, 3 µm resin) at a flow rate of 300 nL min^−1^. Separation of peptides was achieved through chromatographic resolution, followed by mass spectrometric analysis using a Q Exactive mass spectrometer (Thermo Scientific) operated in positive ionization mode.

The mass spectrometric acquisition was performed utilizing a data‐dependent top20 method, selectively targeting the most intense precursor ions (m/z range: 300–1800) from the survey scan for subsequent fragmentation. The automatic gain control (AGC) target was set to 3e6, with a maximum injection time of 50 ms to ensure adequate ion accumulation. A dynamic exclusion window of 60.0 s was implemented to prevent repetitive analysis of the same ions. High‐resolution mass spectrometry was employed for survey scans (resolution of 70000 at m/z 200) and HCD fragmentation spectra (resolution of 17500 at m/z 200), with an isolation width of 2 m/z. The normalized collision energy was set to 30 eV, and the underfill ratio, which dictates the minimum target ion population required to initiate fragmentation, was defined as 0.1%.

### Molecular Docking

The molecular docking models were ANKRD1 (AlphaFold Protein Structure Database: Q15327) and ACSL3 (AlphaFold Protein Structure Database: O95573), sourced from the AlphaFold Database. HDOCK SERVER (http://hdock.phys.hust.edu.cn/) facilitated the docking process, with ACSL3 as the receptor and ANKRD1 as the ligand. Protein preparation was done in PyMOL 2.4, removing water and excess ligands, and adding hydrogens. The model with the lowest binding energy was chosen for further analysis, and PyMOL was used to visualize the interactions.

### TCGA Dataset

TCGA and GTEx datasets (gepia.cancerpku.cn) were queried to validate the prognostic significance of PD‐L1 and Nampt and its expression correlation in CC.

### Statistical Analysis

Statistical analysis was performed using GraphPad Prism 8 software. Quantification data are presented as means ± SD of biological triplicates, and the differences between the two groups were compared using an unpaired two‐tailed Student's t‐test. Survival curves were calculated by the Kaplan–Meier method. *p* < 0.05 was considered statistically significant and asterisks denoted statistical significance (^*^ *p* < 0.05, ^**^ *p *< 0.01, ^***^ *p* < 0.001, ^****^ *p* < 0.0001).

## Conflict of Interest

The authors declare no conflict of interest.

## Author Contributions

X.L. P.J. Q.T. contributed equally to this work. Y.C., D.C., M.Y., X.L. designed this study. Y.C., D.C., M.Y., X.L., P.J., Q.T. supervised this study. M.Z., H.X., L.W., F.X., M.Z. coordinated the sample collection. S.C., Y.Y., J.Z., X.P., J.Z., L.W., Y.C., M.W. assisted in the experiment. X.L., P.J., Q.T. conducted the data analysis. X.L., Y.C., D.C., P.J., Q.T. wrote the original manuscript. X.L., Y.C., D.C., M.Y. reviewed and polished the manuscript.

## Supporting information



Supporting Information

## Data Availability

The data and scripts supporting the findings of this study are available from the corresponding author.
