# Supplementary material for: NAD+ Metabolism Reprogramming Drives SIRT1‐Dependent Deacetylation Inducing PD‐L1 Nuclear Localization in Cervical Cancer
Source: Adv Sci (Weinh). 2025 Feb 23;12(15):2412109. doi: 10.1002/advs.202412109 (PMC12005810; doi:10.1002/advs.202412109)
Supplement: Supplementary file 1 — Supporting Information [file ADVS-12-2412109-s001.docx]

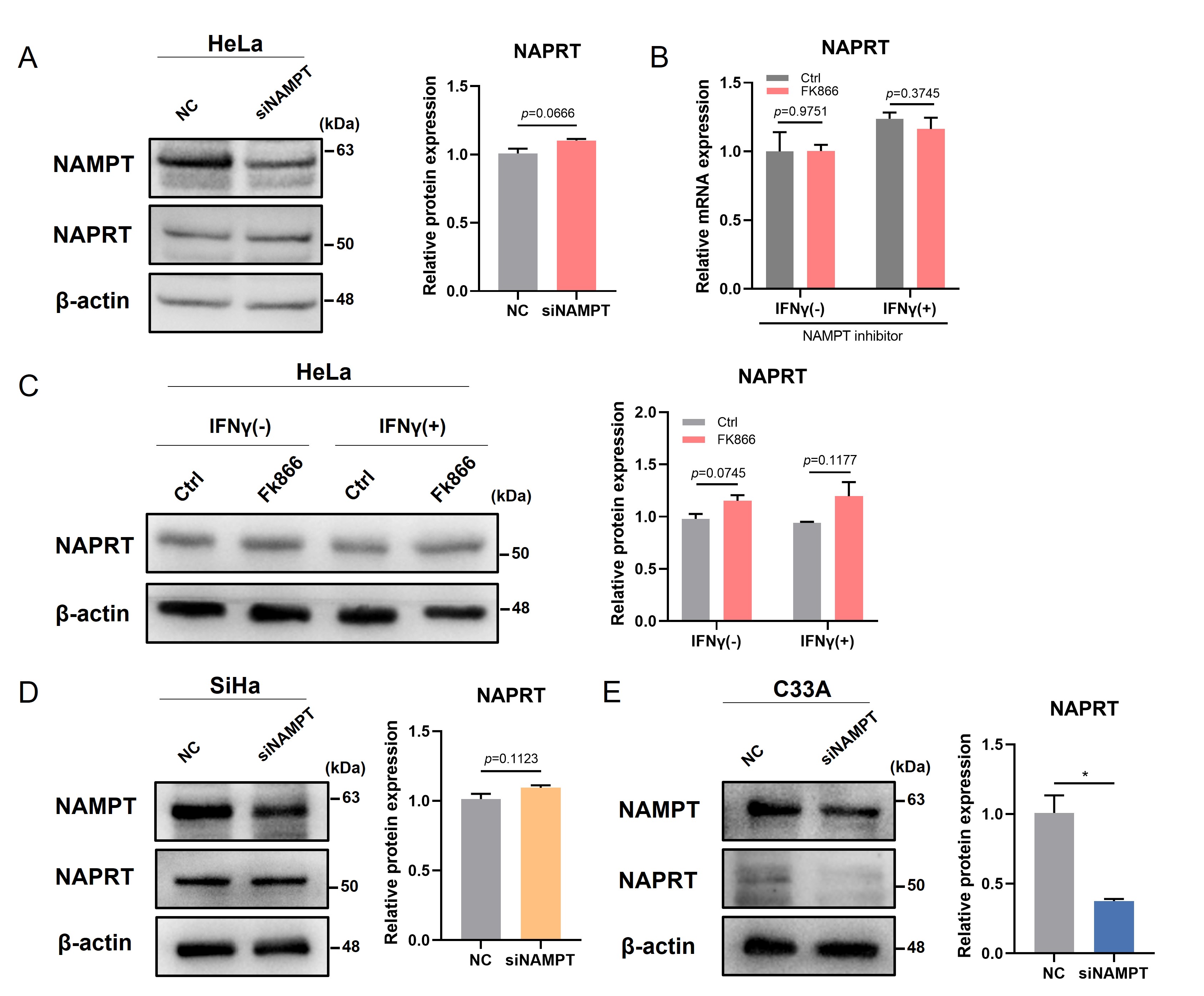


**Fig S1. NAMPT-mediated NAD^+^ metabolism promotes the expression level of PD-L1 in CC cells.**

1. The protein levels of NAMPT and NAPRT in HeLa with Nampt KD. B-C) The mRNA and protein level of NAPRT in HeLa with Nampt inhibitor (FK866) treatment. D-E) The protein levels of NAMPT and NAPRT in SiHa and C33A with Nampt KD.


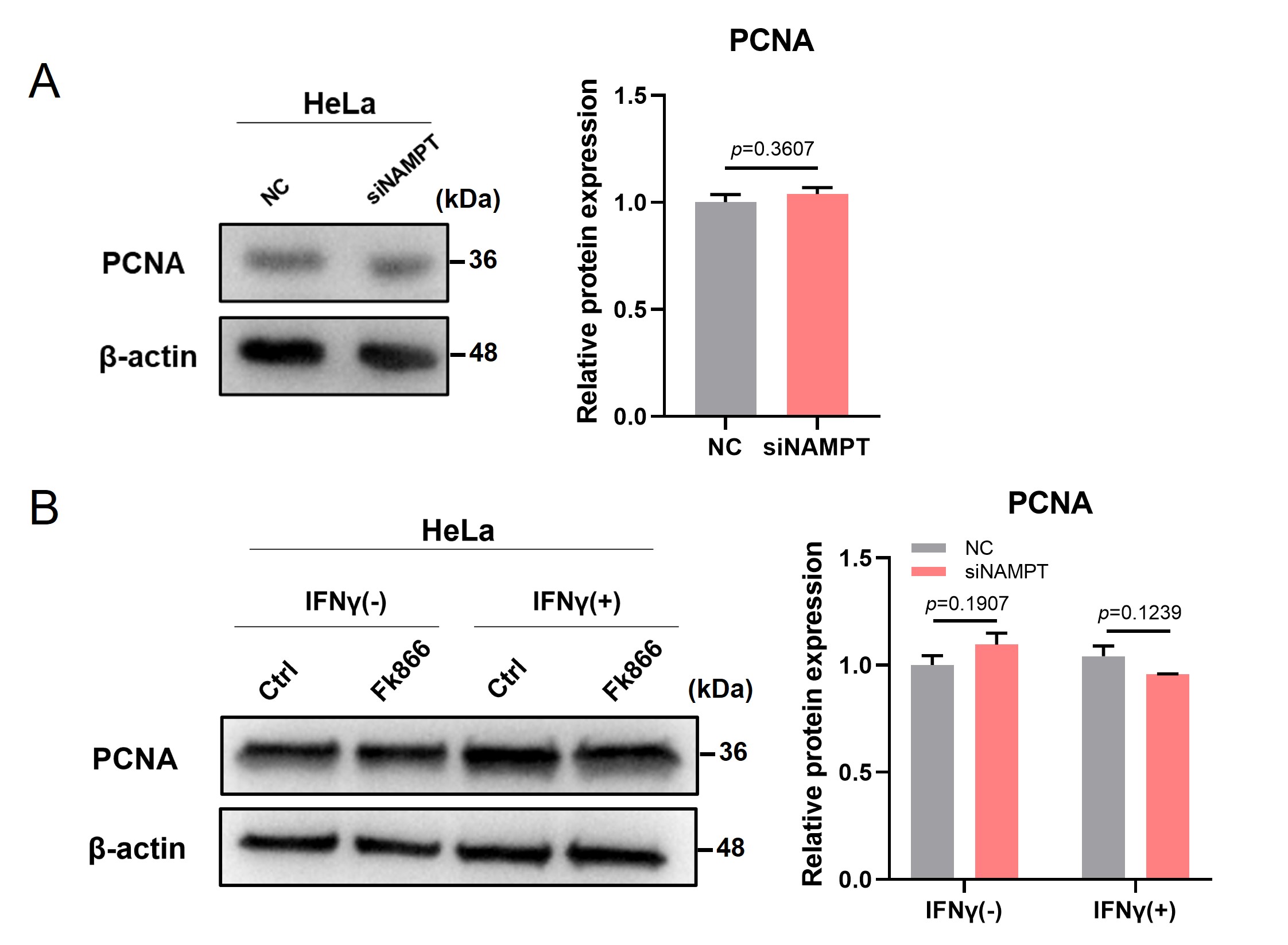


**Fig S2. NAMPT enhances the nuclear localization of PD-L1 in CC cells.**

A) The protein levels of PCNA in HeLa with Nampt KD. B) The protein levels of PCNA in HeLa with Nampt inhibitor FK866 pretreatment upon or without IFNγ stimulation.
